# Measuring Heat Dissipation and Entropic Potential in Battery Cathodes Made with Conjugated and Conventional Polymer Binders Using *Operando* Calorimetry

**DOI:** 10.1021/acsapm.3c02751

**Published:** 2024-05-02

**Authors:** Sun Woong Baek, Charlene Z. Salamat, Rodrigo Elizalde-Segovia, Pratyusha Das, Matevž Frajnkovič, Yucheng Zhou, Barry C. Thompson, Sri R. Narayan, Sarah H. Tolbert, Laurent Pilon

**Affiliations:** † Mechanical and Aerospace Engineering Department, Henry Samueli School of Engineering and Applied Science, University of California, Los Angeles, Los Angeles, California 90095, United States; ‡ Department of Chemistry and Biochemistry, University of California, Los Angeles, Los Angeles, California 90095, United States; § Department of Chemistry and Loker Hydrocarbon Research Institute, University of Southern California, Los Angeles, California 90089, United States; ∥ Department of Materials Science and Engineering, University of California, Los Angeles, Los Angeles, California 90095, United States; ⊥ California NanoSystems Institute, University of California, Los Angeles, Los Angeles, California 90095, United States; # Institute of the Environment and Sustainability, University of California, Los Angeles, Los Angeles, California 90095, United States

**Keywords:** entropic potential, calorimetry, heat generation, conjugated polymers, cathode binders

## Abstract

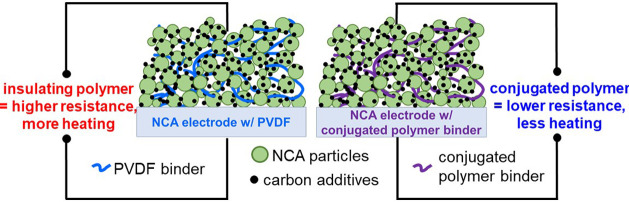

This study explores the influence of electronic and ionic conductivities on the behavior of conjugated polymer binders through the measurement of entropic potential and heat generation in an operating lithium-ion battery. Specifically, the traditional poly(vinylidene fluoride) (PVDF) binder in LiNi_0.8_Co_0.15_Al_0.05_O_2_ (NCA) cathode electrodes was replaced with semiconducting polymer binders based on poly(3,4-propylenedioxythiophene). Two conjugated polymers were explored: one is a homopolymer with all aliphatic side chains, and the other is a copolymer with both aliphatic and ethylene oxide side chains. We have shown previously that both polymers have high electronic conductivity in the potential range of NCA redox, but the copolymer has a higher ionic conductivity and a slightly lower electronic conductivity. Entropic potential measurements during battery cycling revealed consistent trends during delithiation for all of the binders, indicating that the binders did not modify the expected NCA solid solution deintercalation process. The entropic signature of polymer doping to form the conductive state could be clearly observed at potentials below NCA oxidation, however. *Operando* isothermal calorimetric measurements showed that the conductive binders resulted in less Joule heating compared to PVDF and that the net electrical energy was entirely dissipated as heat. In a comparison of the two conjugated polymer binders, the heat dissipation was lower for the homopolymer binder at lower C-rates, suggesting that electronic conductivity rather than ionic conductivity was the most important for reducing Joule heating at lower rates, but that ionic conductivity became more important at higher rates.

## 1 Introduction

Due to their high specific energy and power densities, lithium-ion batteries (LIBs) are, today, the main energy storage devices for electric vehicles and portable electronics.^1−3^ Among the many components of a traditional lithium-ion battery, the binder holds all of the components together in contact with the current collector and prevents the loss of material from dissolution into the electrolyte. Currently, poly(vinylidene fluoride) (PVDF) is the most commonly used binder due to its chemical stability over a wide electrochemical potential window. While flexible backbones such as PVDF help in mechanical adhesion and interconnectivity between the components of a composite electrode, the insulating polymer contributes little else while adding mass to the electrode. In past studies, we have proved that while PVDF is electrochemically stable, it hinders ion transport and is electrically insulating.^4,5^

To increase the overall conductivity of the electrode, conjugated polymers can be used as battery binders in place of traditional insulating polymers, and in fact, conjugated polymers have been widely investigated as both cathode and anode binders.^4−13^ While semiconducting polymers have wide band gaps and are insulating in their neutral forms, during battery cycling, the binder can be electrochemically oxidized (cathode, *p*-type) or reduced (anode, *n*-type) so that it becomes electrochemically doped and conducting. When an electron is removed from the highest occupied molecular orbital (HOMO) by oxidation, the conjugated polymer becomes *p*-doped. Additionally, some semiconducting polymers have the ability to solvate and transport ions along with electrons.^11,14^

In order to achieve multifunctionality in battery binders, we have previously investigated the mixed electron and ion-conducting polymer dihexyl-substituted poly(3,4-propylenedioxythiophene) (PProDOT-Hx_2_) and its derivates with polar oligoether (OE) side chains.^4,5^ Our previous studies demonstrated that the electronic conductivity of PProDOT-Hx_2_ increases significantly with doping.^4^ Furthermore, adding OE side chains to the PProDOT backbone aids in lithium-ion transport, thus increasing ionic conductivity.^5^

The objective of this study is to employ potentiometric entropy and calorimetric measurements to investigate the impact of electronic and ionic conductivities in conjugated polymeric cathode binders on the electrical losses and heat generation within lithium-ion battery cells. Conventional electrochemical characterization techniques and potentiometric entropy measurements were used to investigate the physicochemical phenomena taking place in working electrodes consisting of LiNi_0.8_Co_0.15_Al_0.05_O_2_ (NCA) with different polymer binders. The instantaneous heat generation rate was also measured by *operando* isothermal calorimetry under constant current cycling for the same electrodes and binders. In all cases, 1 M lithium bis(trifluoromethanesulfonyl)imide (LiTFSI) in EC/DMC 1:1 v/v was used as the electrolyte, and Li metal was used as the counter electrode. These measurements were used to shed light on the physicochemical phenomena responsible for any energy dissipation upon cycling.

## 2 Background

### 2.1 Entropic Potential Measurements

Entropic potential measurements consist of measuring the open circuit voltage *U*_OCV_(*x*, *T*) and its derivative with respect to temperature ∂*U*_OCV_(*x*, *T*)/∂*T* as functions of the state of charge or lithium composition *x* at a given temperature *T*. The open circuit voltage *U*_OCV_(*x*, *T*) of a battery cell consisting of an NCA-based working electrode and a metallic Li counter electrode can be written as^15,16^
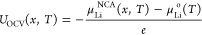
1Here, μ_Li_^NCA^(*x*, *T*) and μ_Li_^o^(*T*) are the Li chemical potentials in the working electrode made of NCA and at the metallic Li counter electrode, respectively, and *e* is the unit charge. Our previous study^16^ derived the relationships between *U*_OCV_(*x*,*T*) and ∂*U*_OCV_(*x*,*T*)/∂*T* and the thermodynamic properties of the LIB materials for lithium intercalation without or with ion ordering in a homogeneous solid solution, first-order phase transition with two-phase coexistence, and first-order phase transitions passing through with a stable intermediate phase. Ignoring the contributions from the surface energy of the metallic lithium counter electrode, ∂*U*_OCV_(*x*, *T*)/∂*T* can be written as
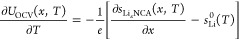
2where *s*_Li_*x*_NCA_ (*x*,*T*) and *s*_Li_^0^(*T*) are the entropy normalized by the number of moles of NCA or the number of moles of metallic Li, respectively. The latter is independent of *x* and was estimated to be 29 J/mol·K at room temperature.^17^ Therefore, the entropic potential ∂*U*_OCV_(*x*,*T*)/∂*T* depends on composition *x* through the partial molar entropy of Li_*x*_NCA, denoted by ∂*s*_Li_*x*_NCA_(*x*,*T*)/∂*x*. As such, measuring ∂*U*_OCV_(*x*, *T*)/∂*T* as a function of *x* can help to identify the physicochemical phenomena occurring in the NCA particles of the working electrode during cycling.

### 2.2 Calorimetry

Various calorimetric measurement techniques have been utilized to measure the thermal energy dissipated and the electrical energy lost in batteries during operation. Techniques such as differential scanning calorimetry,^18,19^ accelerating rate calorimetry,^20,21^ and *operando* isothermal calorimetry^22,23^ have been employed to design thermal management systems and prevent thermal runaway.^20^ They have also been used to gain insights into transport and chemical processes taking place in the cell during cycling.^22,23^ Our previous studies designed, built, and used a custom-made *operando* isothermal calorimeter able to measure the instantaneous heat generation rates^24^ at each electrode during cycling. They have unveiled the thermal signatures of different physicochemical phenomena including ion adsorption/desorption in supercapacitors,^25−28^ ion intercalation,^26^ electrolyte decomposition at high voltages and/or high temperatures,^26,29^ overscreening effect,^29,30^ resistive losses,^23,25−30^ and insulator to metal transition.^23^

The total instantaneous heat generation rate *Q̇*_*T*_(*x*,*T*) (in W) in a battery cell can be expressed as^22,23,31,32^
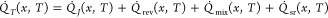
3where *Q̇*_*J*_(*x*,*T*) corresponds to Joule heating, *Q̇*_rev_(*x*,*T*) is the reversible entropic heat generation, *Q̇*_mix_(*x*,*T*) is the so-called enthalpy of mixing, and *Q̇*_sr_(*x*,*T*) is the heat generation due to side reactions. Here, *Q̇_T_*(*x*,*T*) is negative when the battery cell absorbs heat and positive when it releases heat.

Using isothermal conditions, the exothermic Joule heating *Q̇*_*J*_(*x*,*T*) related to irreversible resistive losses can be defined as^22,23,31,32^
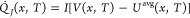
4where *I* is the applied current and [*V*(*x*,*T*)–*U*^avg^ (*x*,*T*)] is the cell overpotential, while *U*^avg^(*x*,*T*) is the open circuit voltage evaluated at the volume-averaged concentration of lithium ions in the cell.^33^ In practice, *U*^avg^(*x*,*T*) is “the potential to which the cell would relax if the current was interrupted”^32^ and can be measured using galvanostatic intermittent titration technique (GITT) at the C-rate used for the calorimetric measurements.

The reversible entropic heat generation rate *Q̇*_rev_(*x*,*T*) can be expressed as^22,31,32^
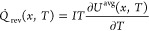
5

Under extremely small current, Li intercalates uniformly in the electrode, and the operating voltage *V*(*x*,*T*) equals the open circuit voltage *U*_OCV_ (*x*,*T*), i.e., *V*(*x*,*T*) = *U*^avg^ (*x*,*T*) = *U*_OCV_ (*x*,*T*). Then, Joule heating vanishes and *Q̇*_rev_(*x*,*T*) is the only cause of heat dissipation.^34^ Conversely, for large current, *Q̇*_*J*_(*x*,*T*) dominates^34^ and Li^+^ concentration gradients become important inside the working electrode and electrolyte due to mass transfer limitations.

The heat generation rate associated with the enthalpy of mixing *Q̇*_mix_(*x*,*T*) arising from ion concentration gradients in the electrodes and electrolyte can be written as^22,31,32^
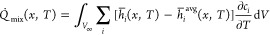
6where *V*_∞_ is the volume of the cell, *h̅*_*i*_ (*x*,*T*) and *h̅*_*i*_^avg^ (*x*,*T*) are, respectively, the local and volume-averaged partial molar enthalpy of ion species *i*, and *c*_*i*_ is the local concentration of the ion species. The enthalpy of mixing primarily arises from four distinct ionic concentration gradients: (i) across the electrode caused by nonuniform current distribution, (ii) within the vacancies of the electrode, (iii) across the electrolyte due to mass transfer resistance, and (iv) within intercalated lithium in the electrode due to electrochemical reactions.^33,35^ Typically, the primary factor contributing to the enthalpy of mixing in LIBs is the ionic concentration gradient of intercalated lithium in the electrode.^32^ Therefore, *Q̇*_mix_(*x*,*T*) should be small if the charging rate is small or if the Li^+^ transport in the electrode is fast.

Most calorimetric analyses of LIBs have overlooked the heat generation attributed to side reactions, *Q̇*_sr_(*x*,*T*), since most undesired side reactions can be avoided by cycling the cell within a suitable potential window and utilizing chemically stable components.^22,31,32,36^ Subsequently, the aging of LIBs takes place gradually, and the associated *Q̇*_sr_(*x*,*T*) is negligibly small at the beginning of the life of LIBs.^22^

The total amount of heat *Q*_*T*_ (in J) released over a cycle as well as those associated with Joule heating *Q*_*J*_ and enthalpy of mixing *Q*_mix_ are expressed as
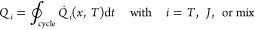
7Note that the reversible heat generation rate *Q̇*_rev_(*x*,*T*) averages to zero over a cycle, i.e., *Q*_rev_ = 0.

Moreover, the net electrical energy losses Δ*E*_*e*_ (in J) over a cycle can be defined as the difference between the electrical energy supplied during charging and that recovered during discharging. Graphically, Δ*E*_e_ corresponds to the area enclosed by the hysteresis described by the cycle plotted in the voltage *V*(*x*, *T*) vs charge transferred *q* diagram, expressed as^22,23^
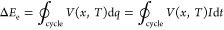
8Here, *I* is the current such that *I* = d*q*/d*t*. Based on the first law of thermodynamics, the net electrical energy loss Δ*E*_e_ equals the total thermal energy *Q*_*T*_ dissipated during a cycle, i.e.,
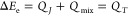
9

This energy balance has been verified experimentally with cells made of TiNb_2_O_7_,^23^ PNb_9_O_25_,^37^ Ti_2_Nb_2_O_9_,^38^ or (W_0.2_V_0.8_)_3_O_7_^39^ electrodes with the Li metal counter electrode in organic electrolytes.

In the present study, entropic potential measurements and *operando* isothermal calorimetry were used to investigate the energy dissipation mechanisms in NCA electrodes made with three different cathodic polymer binders during cycling. The effect of using conjugated polymer binders on heat generation was investigated with (i) dihexyl-substituted poly(3,4-propylenedioxythiophene), referred to as PProDOT-Hx_2_, (ii) a random copolymer, where dihexyl-substituted PProDOT with 25% hexyl side chains replaced with OE side chains and referred to as (75:25) PProDOT, where the 75 refers to the amount of hexyl side chains and 25 is the amount of oligoether (OE) side chains, and (iii) the insulating poly(vinylidene fluoride) (PVDF) used as a reference.

## 3 Materials and Methods

### 3.1 Synthesis of PProDOT-Based Conjugated Polymer Binders

**Scheme 1:**
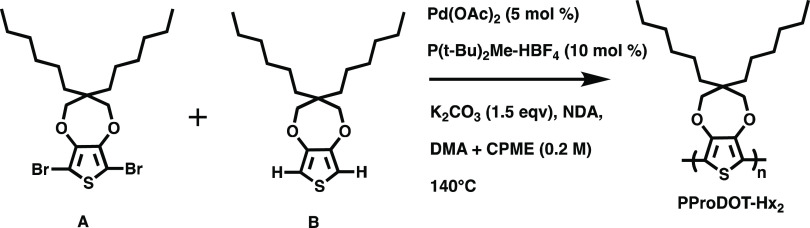
Synthesis of PProDOT-Hx_2_, A Conjugated Polymer Battery Binder^4^

**Scheme 2:**
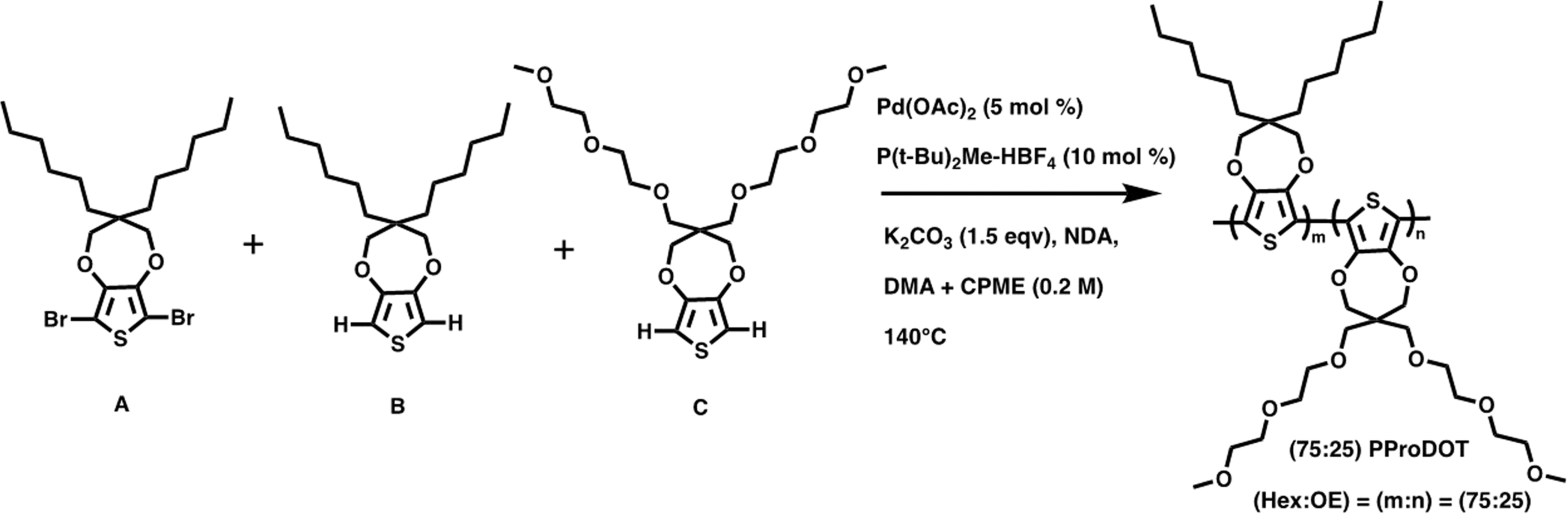
Synthesis of (75:25) PProDOT, Where Molar Ratio of C is 25%, Such That (*m*:*n*) PProDOT of (75:25) Ratio of Side Chains Is Created^5^

All chemical reactions were performed in oven-dried glassware under dry N_2_, unless otherwise noted. Inorganic reagents and solvents were brought from commercial sources through VWR and used as received unless otherwise noted. Anhydrous *N*,*N*-dimethylacetamide (DMA) and cyclopentyl methyl ether (CPME) were purchased from Acros Organics and used as received. Neodecanoic acid (NDA) (Strem Chemicals), P(*t*-Bu)_2_MeHBF_4_ (Sigma-Aldrich), and Pd(OAc)_2_ (>98% TCI) were purchased and used as received. K_2_CO_3_ was dried at 120 °C in a vacuum oven overnight prior to use. Monomers A, B, and C, and polymers PProDOT-Hx_2_ (Scheme 1, *M*_n_ = 17.4 kDa, *Đ* = 1.76) and (75:25) PProDOT (Scheme 2, *M*_n_ = 16.3 kDa, *Đ* = 1.78) were synthesized following the procedures previously reported in the literature.^4,5^

### 3.2 Electrode, Cell Fabrication, and Electrochemical Testing

NCA (LiNi_0.8_Co_0.15_Al_0.05_O_2_, NEI Corporation), Super P carbon black (MTI Corp.), and multiwalled carbon nanotubes (MWCNTs, Cnano, OD × ID × L: 25 nm × 10 nm × 10 μm) powders were gently mixed in a mortar and pestle for 30 min and added to a conjugated polymer in 1,2-dichlorobenzene (ODCB) solution (54 g L^–1^) in a weight ratio of 90:3:3:4, respectively. The resulting slurry was stirred overnight and coated onto aluminum foil using the doctor blade set for a thickness of 35 μm. The electrode film was vacuum-dried for 48 h, roll-pressed (12 in vice-mount slip roll T10727, Grizzly Industrial), and cut onto 14 mm-diameter discs. The control PVDF (MTI Corp.) electrodes were prepared using the same method and weight ratio but by dissolving PVDF in *N*-methyl-2-pyrrolidone (NMP, Sigma-Aldrich). The approximate active material areal mass loading of the different electrodes was 7.7 ± 0.7 mg cm^–2^. Electrodes for the *operando* isothermal calorimetry measurements were cut onto 1 × 1 cm squares, with an active material mass loading of approximately 13.4 ± 0.2, 14.1 ± 0.3, and 10.2 ± 0.8 mg cm^–2^ for NCA–PVDF, NCA–(75:25) PProDOT and NCA–PProDOT-Hx_2_ electrodes, respectively.

Coin cells were fabricated utilizing NCA electrodes as the working electrode with metallic lithium (MTI Corp., *D* × *T*: 16 mm × 0.6 mm) as the counter/reference electrode and Celgard 2325 (PP/PE/PP) 25 μm in thickness as the separator with 1.0 M lithium bis(trifluoromethanesulfonyl)imide in 1:1 ethylene carbonate and dimethyl carbonate (LiTFSI, EC/DMC = 50/50 (v/v), Sigma-Aldrich). The added electrolyte/active material ratio was 8 μL per mg of NCA. CR2032 coin-type cells were assembled and crimped with a pressure-controlled electric crimper machine (MSK-160E, MTI Corp.) between 0.9 and 1 ton in an argon-filled glovebox (VAC systems 60387, NexGen 2P, Vacuum Atmospheres Company) with less than 0.4 ppm of moisture and 0.2 ppm of oxygen. All experiments were performed at room temperature.

Electrochemical characterization and galvanostatic charge–discharge (GCD) cycling were performed by using a high-accuracy potentiostat (Biologic, VSP-300). The C-rate was defined as the reversible capacity of NCA at 1C corresponding to 160 mA g^–1^. The imposed potential window was between 4.2 and 2.7 V vs Li/Li^+^. Specific capacity (mAh g^–1^) was based on the weight of the active material (NCA) as the capacity contribution from the conductive polymer was negligible.

### 3.3 Potentiometric Entropy Measurements

The open circuit voltage *U*_OCV_(*x*,*T*) and the entropic potential ∂*U*_OCV_(*x*,*T*)/∂*T* of the coin cells were measured as functions of lithium composition *x* using the potentiometric entropy measurement technique and the apparatus described previously^17,23^ and in Figures S1 and S2. The measurements were performed by imposing current pulses for approximately 30 min equivalent to a C-rate of C/10. The pulses were followed by a relaxation period of approximately 3 h when the temperature was varied between 15 to 25 °C in 5 °C increments, as described in detail in ref (36). The lithium composition *x* in Li_*x*_NCA can be estimated based on the charging/discharging time, *t* (in s), i.e.,
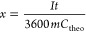
10Here, *m* is the mass loading of the active material in the electrode and *C*_theo_ is the theoretical capacity of NCA calculated as *C*_theo_ = 160 mAh g^–1^ derived from one electron per transition metal. To ensure that the coin cell had reached thermodynamic equilibrium, we confirmed that (i) the temperature difference between the cold plate and the top of the coin cell surface was less than 0.1 °C and (ii) ∂*U*^avg^(*x*,*T*)/∂*T* was below 5 mV/h. In addition, *U*^avg^(*x*,*T*) and ∂*U*^avg^(*x*,*T*)/∂*T* were also measured using the same procedure and the same relaxation time as that used for measuring *U*_OCV_(*x*,*T*) but with current pulses corresponding to C-rates of 1C, 2C, and 3C.

### 3.4 Operando Isothermal Calorimetry

The instantaneous heat generation rates at the bare NCA working electrode and at the metallic lithium counter electrode were measured individually during galvanostatic cycling using a custom-made isothermal calorimeter described previously^24^ and in Figures S3 and S4. The calorimetric cell consists of (i) a 1 × 1 cm^2^ NCA-based electrode with PVDF, PProDOT-Hx_2_, or (75:25) PProDOT binders. The working electrodes had a mass ratio of 90:3:3:4 of NCA: super P: carbon nanotube (CNT): binder. (ii) two 50 μm-thick Celgard C380 polypropylene/polyethylene separator sheets, (iii) 1 M LiTFSI in EC/DMC 1:1 v/v (Sigma-Aldrich) as the electrolyte, and (iv) 1 × 1 cm^2^ polished metallic lithium (Sigma-Aldrich) as the counter electrode. Table 1 summarizes the mass loading of NCA in electrodes made with three different cathodic polymer binders. As discussed in ref (24), the instantaneous heat generation rate *Q̇*_i_(*t*) (in mW) at each electrode was computed with the thermoelectric heat flux sensors in direct thermal contact with the back of the current collector, i.e.,^24^
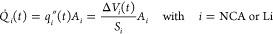
11Here, *A*_*i*_ = 1 × 1 cm^2^ is the footprint area of the heat flux sensor, Δ*V*_*i*_ and *S*_*i*_ denote the measured voltage (in V) and the sensitivity (in μV/(W/cm^2^)) of the heat flux sensor, respectively. The total instantaneous heat generation rate in the entire calorimetric cell is given by the sum of the heat generation rate measured at each electrode, i.e., *Q̇*_*i*_(*t*) = *Q̇*_Li_ (*t*) + *Q̇*_NCA_ (*t*).

**Table 1:** Mass Loading of LiNi_0.8_Co_0.15_Al_0.05_O_2_ (NCA) Used in the Working Electrode with Different Binders for Calorimetry Measurements, the Peak Electronic, Ionic Conductivity, and Specific Capacity at 1C from Refs (4,5), and (10)

binder	mass loading of NCA, *m* (mg)	electronic conductivity (S·cm^–1^)	ionic conductivity (S·cm^–1^)	specific capacity at 1C (mAh/kg)
PVDF	13.4			75
PProDOT-Hx_2_	10.2	1	1 × 10^–7^	120
(75:25)PProDOT	14.1	2.4 × 10^–1^	2.8 × 10^–7^	125

Furthermore, the instantaneous heat generation rate *Q̇*_*i*_(*t*) at each electrode “*i*” can be divided into an irreversible *Q̇*_irr,i_(*t*) and a reversible *Q̇*_rev,*i*_ (*t*) contribution, i.e.,
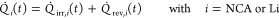
12where *Q̇*_irr,*i*_ (*t*) may vary with time due to potential changes in the electrical and/or ionic conductivities of the electrode during charging/discharging. By definition, the reversible heat generation rate *Q̇*_rev,*i*_ (*t*) averaged over an entire cycle should yield zero, as previously discussed. Thus, the time-averaged irreversible heat generation rate, , at electrode “*i*” can be calculated according to
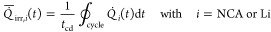
13where *t*_*cd*_ is the cycle period.

## 4 Results and Discussion

### 4.1 *U*_OCV_ (*x*, *T*) and ∂*U*_OCV_ (*x*, *T*)/∂*T* Measurements

**Figure 1:**
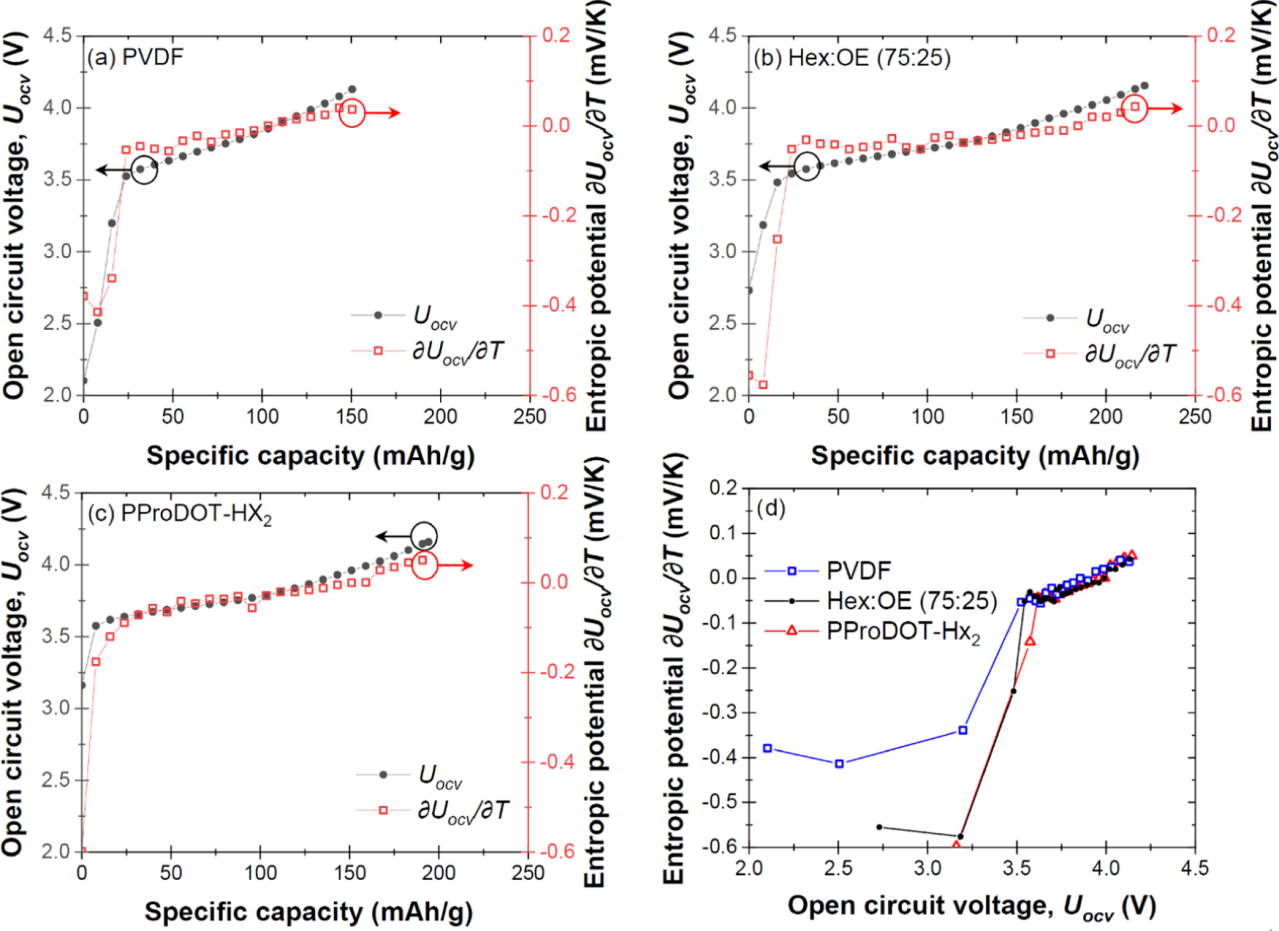
Open circuit voltage *U*_OCV_(*x*,*T*) and entropic potential ∂*U*_OCV_(*x*,*T*)/∂*T* of the cell containing electrodes made of NCA with (a) PVDF, (b) (75:25) PProDOT, and (c) PProDOT- Hx_2_ binders during the first delithiation as functions of specific capacity at temperature *T* = 20 °C; (d) entropic potential ∂*U*_OCV_(*x*,*T*)/∂*T* of all three cells as a function of open circuit voltage, *U*_OCV_(*x*,*T*) during delithiation/charging.

Figure 1a–c plot the open circuit voltage *U*_OCV_(*x*,*T*) and entropic potential ∂*U*_OCV_(*x*,*T*)/∂*T* of the cells containing electrodes made of NCA with (a) PVDF, (b) (75:25) PProDOT, and (c) PProDOT-Hx_2_ binders, measured at 20 °C as functions of specific capacity at C-rate of C/10 during the first delithiation. First, the NCA electrode with (75:25) PProDOT was able to accommodate more lithium than other electrodes and featured the largest specific capacity, while that with PVDF exhibited the smallest specific capacity. This result was consistent with the galvanostatic cycling data reported in our previous study.^5^ In fact, the electronic conductivity of both PProDOT-Hx_2_ and (75:25) PProDOT are orders of magnitude larger than that of PVDF, thus enhancing the electrochemical performance of the electrode.^4,5^Figure 1d indicates that the entropic potential ∂*U*_OCV_(*x*,*T*)/∂*T* is much more negative for the two conjugated polymers PProDOT-Hx_2_ and (75:25) PProDOT than for PVDF when *U*_OCV_(*x*,*T*) ≤ 3.5 *V*. This is reflective of the redox process in the semiconducting polymers, where the polymers are gradually doped and the counterions are migrating into the polymer network to balance the charge on the polymer. The open circuit voltage of the conjugated polymers (∼2.8 V vs Li/Li^+^) pins the open circuit voltage of those cells at ∼2.8 V. However, PVDF is an insulator and remains unchanged during this process, so that NCA surface defects dictate the open circuit voltage of the NCA–PVDF electrode. For *U*_OCV_(*x*,*T*) > 3.5 *V*, ∂*U*_OCV_(*x*,*T*)/∂*T* shows a slow increase with increasing capacity, and the trend is similar for all three polymer binders because it is dictated by NCA oxidation.

**Figure 2:**
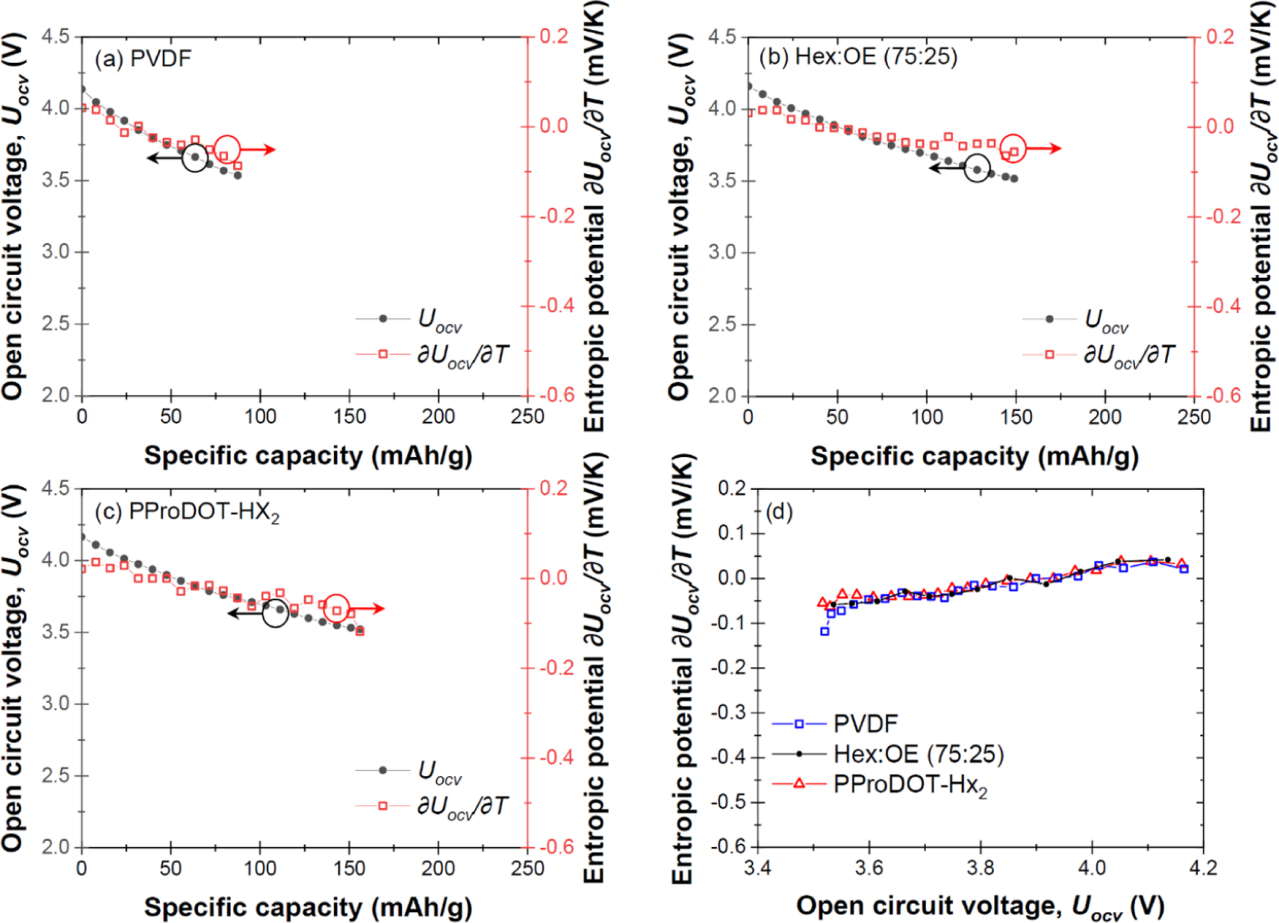
Open circuit voltage *U*_OCV_ (*x*,*T*) and entropic potential ∂*U*_OCV_ (*x*,*T*)/∂*T* as functions of specific capacity at temperature *T* = 20 °C for the cells containing electrodes made of NCA with (a) PVDF, (b) (75:25) PProDOT, and (c) PProDOT-Hx_2_ binders during the first lithiation (following the first delithiation illustrated in Figure 1); (d) entropic potential ∂*U*_OCV_ (*x*,*T*)/∂*T* of all three cells as functions of open circuit voltage *U*_OCV_ (*x*,*T*) during lithiation.

Figure 2a–c plot the open circuit voltage *U*_OCV_(*x*,*T*) and entropic potential ∂*U*_OCV_(*x*,*T*)/∂*T* of the cells containing electrodes made of NCA with (a) PVDF, (b) (75:25) PProDOT, and (c) PProDOT-Hx_2_ binders measured at 20 °C as functions of specific capacity at C-rate of C/10 during the first lithiation, immediately following the delithiation shown in Figure 1. Here also, the NCA electrode with PVDF featured the lowest specific capacity, confirming that the lack of electronic conductivity in PVDF diminished the electrochemical performance of the electrode. The entropic potential ∂*U*_OCV_ (*x*,*T*)/∂*T* of all three cells as a function of open circuit voltage *U*_OCV_(*x*,*T*) in Figure 2d establishes that ∂*U*_OCV_(*x*,*T*)/∂*T* for all three NCA electrodes with different binders were identical across the entire potential window with *U*_OCV_(*x*,*T*) between 3.5 and 4.2 V (Figure 1d).

This result confirms that the differences in ∂*U*_OCV_ (*x*,*T*)/∂*T* for *U*_OCV_ (*x*,*T*) during the first delithiation for *U*_OCV_ (*x*,*T*) ≤ 3.5 V vs Li/Li^+^ could be attributed to initial specific capacity loss from trapped lithium ions and/or activation of the conductive binder through redox reaction(s).

### 4.2 Calorimetric Measurements

**Figure 3:**
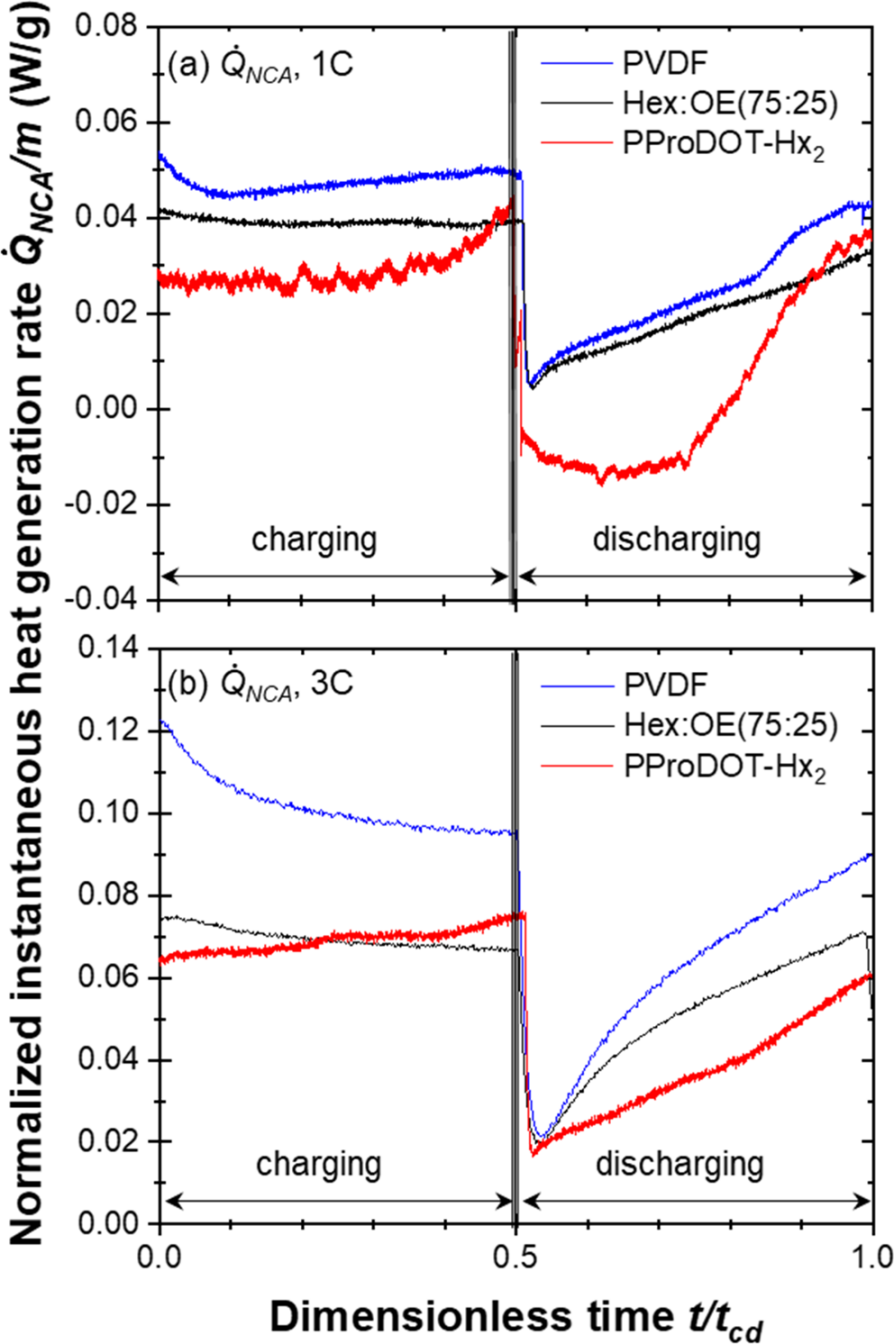
Mass normalized instantaneous heat generation rates *Q̇*_NCA_/*m* at the NCA averaged over five consecutive cycles as a function of dimensionless time *t*_cd_ with the potential window ranging from 2.7 to 4.2 V vs Li/Li^+^ at a C-rate of (a) 1C and (b) 3C.

Figure S5 shows the instantaneous heat generation rates *Q̇*_NCA_ (*t*) measured at the electrodes made of NCA with (a, b) PVDF, (c, d) (75:25) PProDOT, and (e, f) PProDOT-Hx_2_ binder and *Q̇*_Li_ (*t*) measured at the lithium metal counter electrode and averaged over 5 consecutive cycles as functions of dimensionless time *t*/*t*_cd_ at 20 °C and at C-rate of (a–c) 1C and (d–f) 3C. Here, *t*_cd_ is the cycling period starting with charging (delithiation) followed by discharging (lithiation). For both cells containing electrodes made of NCA with (75:25) PProDOT and PProDOT-Hx_2_, *t*_cd_ averaged over 5 consecutive cycles was around 1 h and 50 min, while it was 1 h and 30 min for the cell with an electrode made of NCA with PVDF at C-rate of 1C. Interestingly, *t*_cd_ decreased rapidly at a C-rate of 3C and was measured to be 19 min for the cell containing electrodes made of NCA with PVDF. However, *t*_cd_ was 30 and 32 min for the cell containing electrodes made of NCA with (75:25) PProDOT and PProDOT-Hx_2_, respectively. This difference could be attributed to the fact that the ionic conductivity of both PProDOT-Hx_2_ and (75:25) PProDOT are orders of magnitude larger than that of PVDF, thus enhancing the mobility of ions, enabling the fast ion transport leading to superior electrical performance at higher C-rate. The magnitude of *Q̇*_NCA_ (*t*) at the NCA electrode with PProDOT-Hx_2_ was smaller than those at the NCA electrodes with PVDF and (75:25) PProDOT due to the smaller mass loading (Table 1). Thus, Figure 3 shows the mass normalized instantaneous heat generation rate, *Q̇*_NCA_/*m*, measured at the electrodes made of NCA with PVDF (blue trace), (75:25) PProDOT, (black trace) and PProDOT-Hx_2_ binder (red trace) at C-rate of (a) 1C and (b) 3C, showing that regardless of the C-rate, PVDF consistently had the largest *Q̇*_NCA_/*m*, while PProDOT-Hx_2_ had the lowest. It is also worth noting that during charging, Q̇_NCA_/m remains fairly constant for all three polymers, but during discharging, *Q̇*_NCA_/*m* increases for all three polymers. These behaviors could be attributed to the fact that (75:25) PProDOT and PProDOT-Hx_2_ feature relatively high electronic conductivity compared with the insulating PVDF, thus reducing Joule heating.

**Figure 4:**
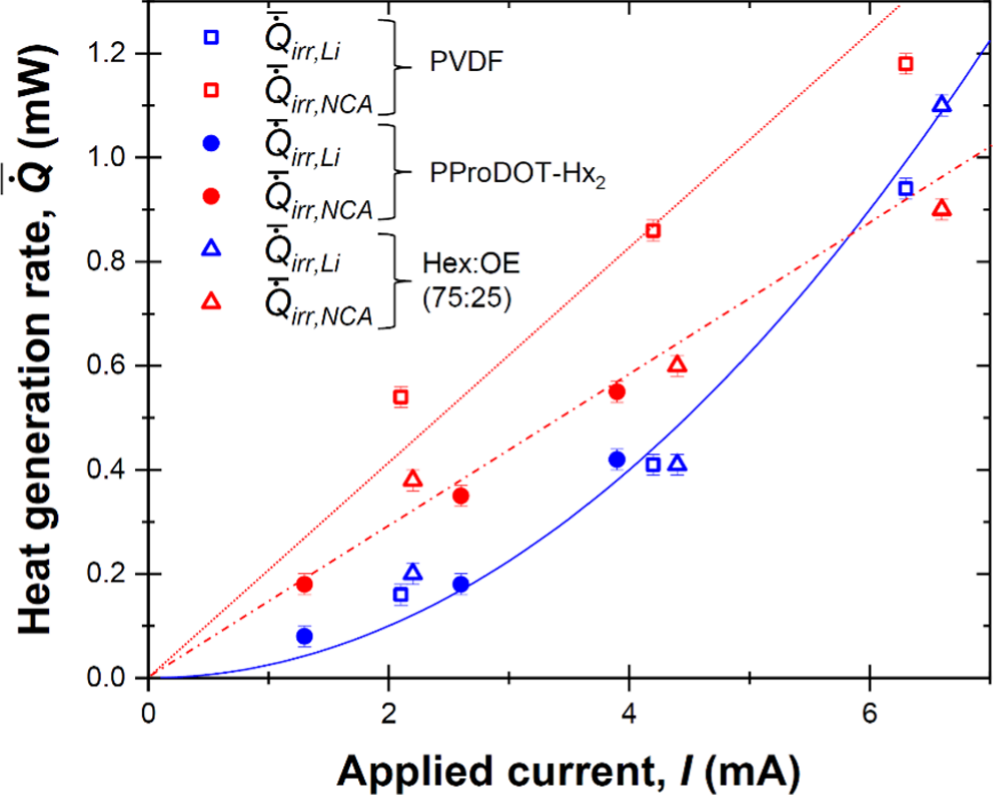
Time-averaged irreversible heat generation rates  at the NCA electrode with different binders and  at the Li metal electrode, as a function of applied current, *I*, based on the isothermal operando calorimetry measurements at temperature *T* = 20 °C (Figure 3).

Figure 4 plots the time-averaged irreversible heat generation rates  and  averaged over five consecutive cycles. The error bars represent two standard deviations or 95% confidence intervals corresponding to 0.015 mW. Fitting of  with respect to the imposed current *I* at the Li metal electrode yields . In other words, the irreversible heat generation was dominated by Joule heating, and the resistance of the metallic Li electrode was constant. By contrast,  increased linearly with respect to applied current *I* for all three different binders. This could be due, in part, to the fact that the electrical resistivity of NCA changes upon lithium intercalation/deintercalation. Moreover, as the C-rate increased, the capacity of the cells with electrodes consisting of NCA with different binders decreased, indicating that the amount of lithium intercalating/deintercalating also decreased. Thus, at high C-rates, the material underwent a narrower change in composition so that the average electrical resistivity also varied with the C-rate. In fact, there was no significant difference between  in the NCA electrodes with PProDOT-Hx_2_ and with (75:25) PProDOT binder as their electrical conductivities are nearly identical.^5^ However,  in the electrode consisting of NCA with PVDF binder was larger than in the electrodes consisting of NCA with PProDOT-Hx_2_ and NCA with (75:25) PProDOT at any C-rate. This behavior could be attributed to the fact that both PProDOT-Hx_2_ and (75:25) PProDOT are conductive binders that enhance the electrical conductivity of the electrodes while PVDF is an insulating binder.^4,5^ These results indicate that electrodes composed of conductive binders are energetically more efficient and result in less electrical loss dissipated as heat.

**Figure 5:**
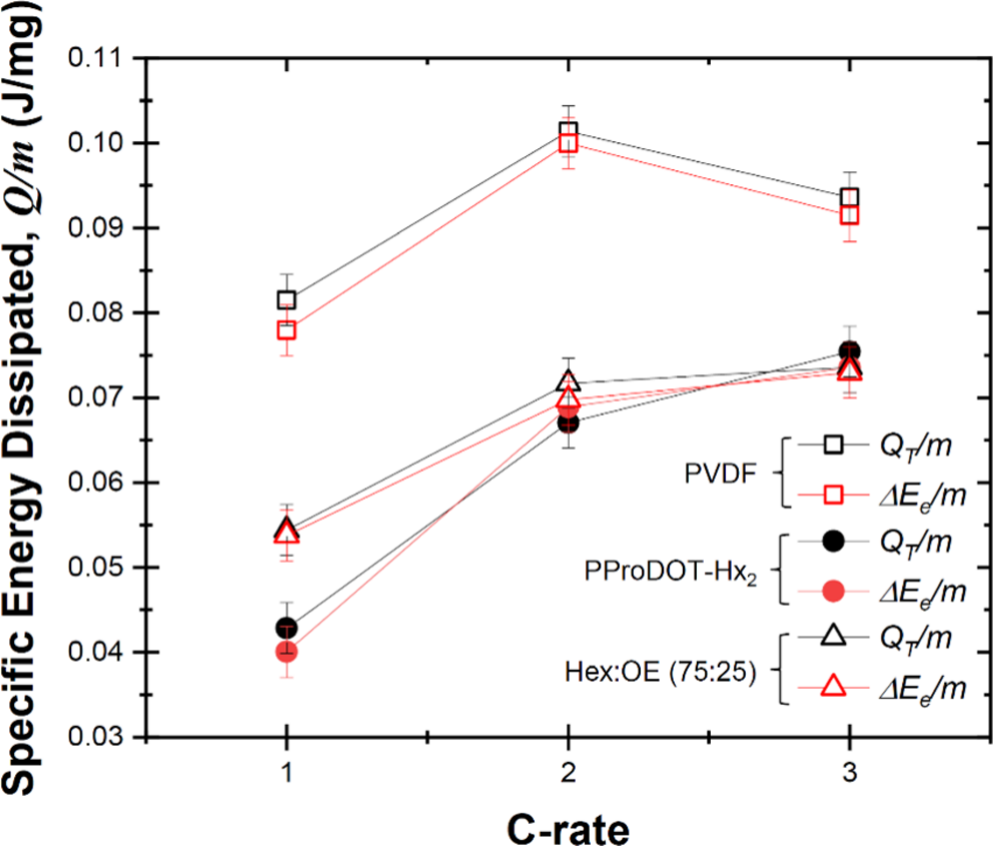
Specific net electrical energy loss Δ*E*_e_/*m* and specific total energy dissipated *Q*_T_/*m* over a charging/discharging cycle for calorimetric cells with NCA electrodes with PVDF, (75:25) PProDOT, and PProDOT-Hx_2_ binders as functions of various C-rates.

Figure 5 plots the net electrical energy loss Δ*E*_e_/*m* and the total thermal energy dissipated *Q*_*T*_/*m* per unit mass of NCA material over a charging/discharging cycle for calorimetric cells with NCA electrodes with PVDF, (75:25) PProDOT, and PProDOT-Hx_2_ as a function of various C-rates. Here, Δ*E*_e_ and *Q*_*T*_ were measured independently using the previously described potentiostat and calorimeter, respectively. First, regardless of the binder, all of the resistive losses were dissipated as heat, i.e., Δ*E*_e_/m ≈ *Q*_*T*_/*m*. Furthermore, the heat dissipated over a cycle *Q*_*T*_/*m* measured for the cell made with NCA and PVDF as the binder was significantly larger than those measured for NCA electrodes made with either PProDOT-Hx_2_ or (75:25) PProDOT binders for any given C-rate. This behavior is attributed to the fact that (75:25) PProDOT and PProDOT-Hx_2_ binders have relatively high electronic conductivity,^4,5^ thus reducing the Joule heating, while PVDF is an insulating binder that impairs electrical connectivity, leading to larger Joule heating, as previously discussed. For the cell made with the PVDF binder, the specific total thermal energy dissipated, *and Q*_*T*_/*m* first increased and then decreased for C-rate ≥2C. By contrast, *Q*_*T*_/*m* increased monotonically with the C-rate for the cells with (75:25) PProDOT and PProDOT-Hx_2_. These observations can be attributed to the fact that the total capacity decreased more significantly with increasing C-rate for the NCA cell made with the PVDF binder compared with those consisting of NCA electrodes with (75:25) PProDOT and PProDOT-Hx_2_ binders. With a smaller total capacity, the total heat generation also decreases.

The trends within the two conducting polymer binders are somewhat more subtle but can also be readily understood. In previous studies, we found that the ionic and electronic conductivities of PProDOT-Hx_2_ and Hex:OE binders increase when electrochemically doped. The peak conductivities measured at room temperature are reproduced in Table 1. The ionic conductivity of PProDOT-Hx_2_ reaches a maximum of 1 × 10^–7^ S·cm^–1^. Adding the OE side chains to the conjugated polymer binder aids in lithium-ion diffusion and in fact doubles the ionic conductivity to 2.8 × 10^–7^ S·cm^–1^.^4,5^ However, the addition of these OE side chains also results in the decrease of electronic conductivity from 1 S·cm^–1^ for PProDOT-Hx_2_ to 0.24 S·cm^–1^ for Hex:OE.^4,5^ Combining these past findings with the net electrical energy loss Δ*E*_e_/*m* and the total thermal energy dissipated *Q*_*T*_/*m* in Figure 5 shows some interesting trends. At a low C-rate (1C), the NCA electrode with PProDOT-Hx_2_ featured significantly lower energy dissipation, indicating that electronic conductivity is the more important factor for heat dissipation than ionic conductivity at these slower rates. At a higher C-rate of 2C, however, Δ*E*_e_/*m* and *Q*_*T*_/*m* converged for both conjugated polymers, but at 3C, electrodes made with the (75:25) PProDOT binder actually had slightly lower heat generation, suggesting that ionic conductivity becomes more important relative to electronic conductivity at higher rates.

## 5 Conclusions

While entropic potential and calorimetry experiments have previously been utilized to investigate energy losses and heat dissipation in battery cells with different active materials, this study focuses on understanding the effect of electronic and ionic conductivities of conjugated polymeric cathode binders on entropic potential, electrical losses, and heat generation in battery cells. To do so, the traditional PVDF binder was replaced with semiconducting polymer binders, either PProDOT-Hx_2_ or copolymer (75:25) PProDOT. These two binders were chosen for their higher electronic and ionic conductivities than PVDF. Specifically, PProDOT-Hx_2_ has a higher electronic conductivity than (75:25) PProDOT while (75:25) PProDOT has a higher ionic conductivity.

This study demonstrated that for all polymer binders, from the insulating PVDF to the conjugated PProDOT-Hx_2_ and its derivative (75:25) PProDOT, the entropic potential values were similar. For delithiation, we observed consistently that both *U*_OCV_(*x*,*T*) and ∂*U*_OCV_(*x*,*T*)/∂*T* increase with increasing specific capacity regardless of the polymer binder used. This shows that the delithiation from the cathode material does not cause significant structural changes or phase transitions. This solid solution deintercalation process ensures that the battery material remains stable, and processes are reversible during cycling. This is a crucial attribute for the long-term performance and cycle life of lithium-ion batteries. Additionally, we observed that the ∂*U*_OCV_(*x*,*T*)/∂*T* at the first lithiation was identical for all of the electrodes but not for the first delithiation, indicating that the specific capacity loss from the first delithiation could result from trapped ions and/or the redox reaction occurring at the semiconducting polymer binders.

Finally, *operando* isothermal calorimetric measurements on NCA/Li metal battery electrodes with different binders established that the use of conductive binders PProDot-Hx_2_ and (75:25) PProDOT resulted in lower electrical losses and heat dissipation, particularly at high C-rates compared to the insulating binder (PVDF) due to their higher electronic and ionic conductivities. Between the two conjugated polymer binders, the heat dissipation was slightly lower for the polymer binder with higher electronic conductivity and lower ionic conductivity, suggesting that electronic conductivity is the most important factor for reducing Joule heating, particularly at higher C-rates.

## Supporting Information Available

Schematic of the experimental setups for the potentiometric entropy and isothermal calorimetry measurements; Measured instantaneous heat generation rates at the NCA made with the three polymeric binders investigated and at the lithium metal electrodes cycled at 1C, 2C, and 3C (PDF)

## Notes

^○^ S.R.N. deceased May 24, 2023.
