# Supplementary material for: Measuring Heat Dissipation and Entropic Potential in Battery Cathodes Made with Conjugated and Conventional Polymer Binders Using Operando Calorimetry
Source: ACS Appl Polym Mater. 2024 May 2;6(9):4954–63. doi: 10.1021/acsapm.3c02751 (PMC11091854; doi:10.1021/acsapm.3c02751)
Supplement: Supplementary file 1 — ap3c02751_si_001.pdf [file ap3c02751_si_001.pdf]

# - Supporting Information -

Measuring Heat Dissipation and Entropic Potential in Battery Cathodes made with Conjugated and Conventional Polymer Binders using Operando Calorimetry

Sun Woong Baek[‡,a], Charlene Z. Salamat[‡,b], Rodrigo Elizalde-Segovia[c], Pratyusha Das[c], Matevž Frajnkovič[a], Yucheng Zhou[a], Barry C. Thompson[c], Sri R. Narayan[c,^], Sarah H. Tolbert[*,b,d,e], Laurent Pilon[*,a,e,f]

‡ These authors contributed equally to this work.

a Mechanical and Aerospace Engineering Department, Henry Samueli School of Engineering and Applied Science, University of California, Los Angeles, Los Angeles, CA 90095, USA

b Department of Chemistry and Biochemistry, University of California, Los Angeles, Los Angeles, CA 90095, USA

c Department of Chemistry and Loker Hydrocarbon Research Institute, University of Southern California, Los Angeles, CA 90089, USA

d Department of Materials Science and Engineering, University of California, Los Angeles, Los Angeles, CA 90095, USA

e California NanoSystems Institute, University of California, Los Angeles, Los Angeles, CA 90095, USA

f Institute of the Environment and Sustainability, University of California, Los Angeles, Los Angeles, CA 90095, USA

* corresponding authors pilon@seas.ucla.edu (L. Pilon) and tolbert@chem.ucla.edu (S. H. Tolbert)

Figure S1 shows the schematic of the experimental setup for the potentiometric entropy measurement. Potentiostat (Biologic, VSP-300) with accuracy of < ±1mV±0.03%, proportional-integral-derivative (PID) temperature controller (TE technology, TC-720), and data acquisition system (Keysight, Agilent 34972A) with accuracy of ± 0. 1 μV were connected to the computer. The data acquisition system collected the temperature readings using the k-type thermocouple (OMEGA, GG-KI-24S-200) at the cold plate, and the top of the coin cell and the collected data were sent to the computer.

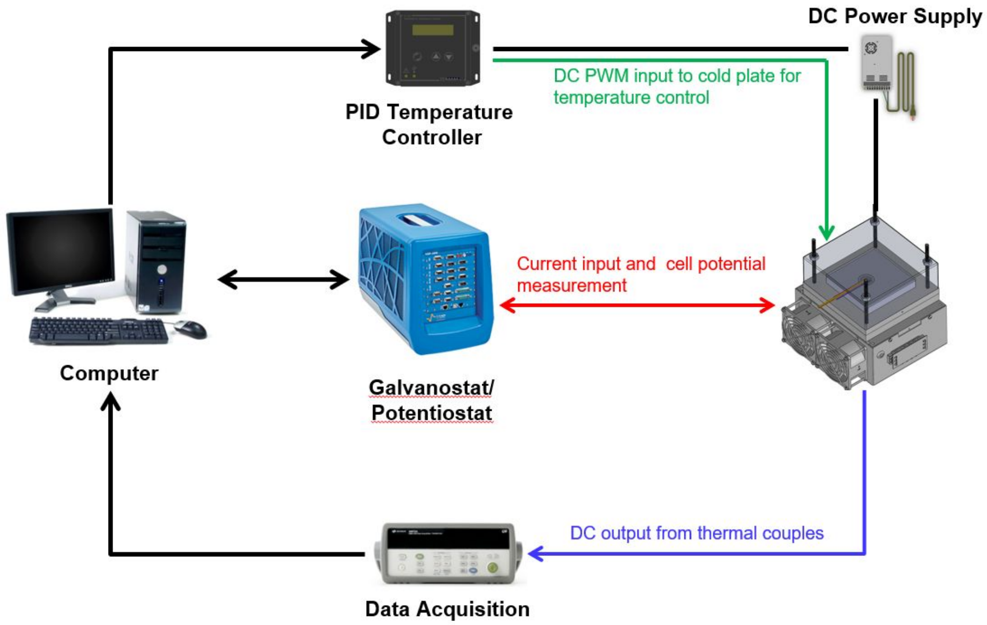

Figure S1. Schematic of the experimental setup for the potentiometric entropy measurement. Reproduced or adapted with permission from Ref.[23]. Copyright 2021 Elsevier.

Figure S2 shows the schematic of the potentiometric entropy measurement device. The coin cell and current collectors are wrapped with Kapton tape to avoid electrical contact with the thermoelectric cold plate. Thermal paste (OMEGA™, OT-201-16) was applied between the thermoelectric cold plate and the wrapped coin cell for better thermal contact. In addition, the thermal paste was also applied between the thermocouple and the coincell and the top was wrapped with copper tape for accurate temperature measurement. The whole coin cell setup was insulated by the styrofoam to avoid any other heat exchange with surrounding for precise temperature control of the coin cells.

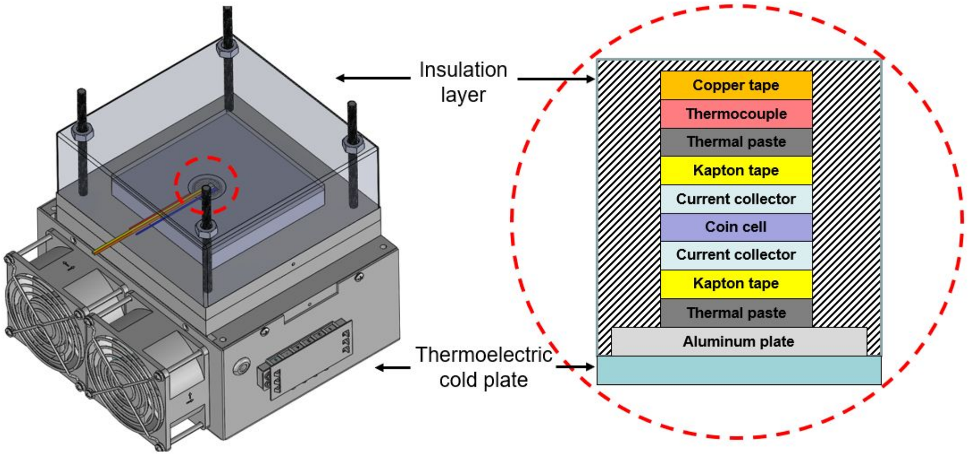

Figure S2. Computer aided design (CAD) model and schematic of the major components of the potentiometric entropy measurement. Reproduced or adapted with permission from Ref.[23]. Copyright 2021 Elsevier.

Figure S3 schematically shows the experimental isothermal calorimeter consisting of (i) two thermoelectric heat flux sensors connected to (ii) a data acquisition (DAQ) system (Keysight, Agilent 34972A), (iii) two identical instrumented cold plates fed with a circulating deionized water from (iv) a temperature-controlled chiller (Cole-Parmer, Polystat), (v) two flow meters (Omega, FLR-1012), and (vi) an electrochemical test section containing a two-electrode cell immersed in an electrolyte and connected to (vii) a potentiostat (Biologic, SP 150). The voltage measured by the DAQ reading the heat flux sensors featured accuracy of ± 0.1 μV. A vertical clamp was used to hold the electrochemical test section and the cold plates together and to ensure good thermal contacts among them. Finally, the entire calorimeter and the cold plates were wrapped in 13 mm thick thermal insulation (Morgan Thermal Ceramics, Ceramic fiber).

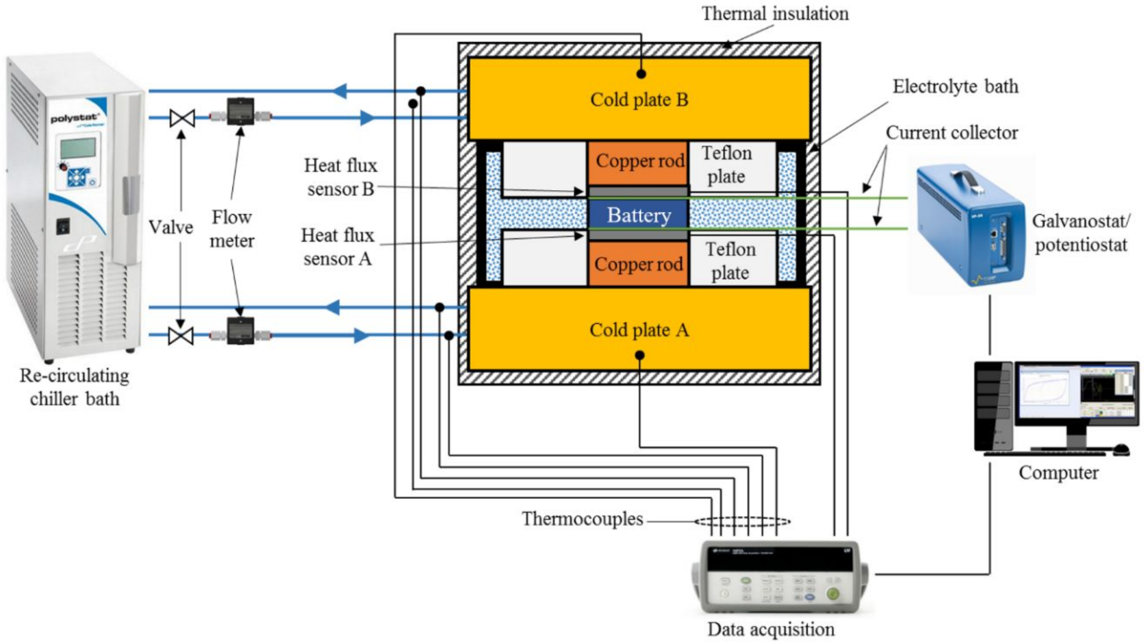

Figure S3. Schematic of an isothermal calorimeter apparatus. Reproduced or adapted with permission from Ref.[27]. Copyright 2018 Elsevier.

Figure S4 shows the schematic of the isothermal calorimeter. The electrochemical test section consisted of two heat flux sensor plates and a cylindrical container made of polytetrafluoroethylene (PTFE) or Teflon. Each heat flux sensor plate consisted of (i) 10 × 10 mm thermoelectric heat flux sensor (GreenTEG, gSKIN-XP) with high accuracy (±10 μW) and with uncertainty under 3%, 0.5 mm in thickness, in thermal contact with (ii) a cylindrical copper rod, 15.9 mm in diameter and 19.5 mm in length, embedded in the center of (iii) a PTFE disc and flush with its surfaces. The thickness and diameter of the PTFE disc were 20 mm and 85 mm, respectively. The copper rod was used to conduct the heat generated in the electrode through the heat flux sensor to the cold plate, maintained at constant temperature. The two heat flux sensor plates were packaged in a 7.5 mm thick PTFE cylinder with height and outer diameter of 40 mm and 100 mm, respectively. Each plate was sealed using a chemical resistance O-ring gasket (MSCdirect, Viton-235) 3.5 mm in thickness and 86 mm in outer diameter to prevent electrolyte evaporation and interaction with surrounding air.

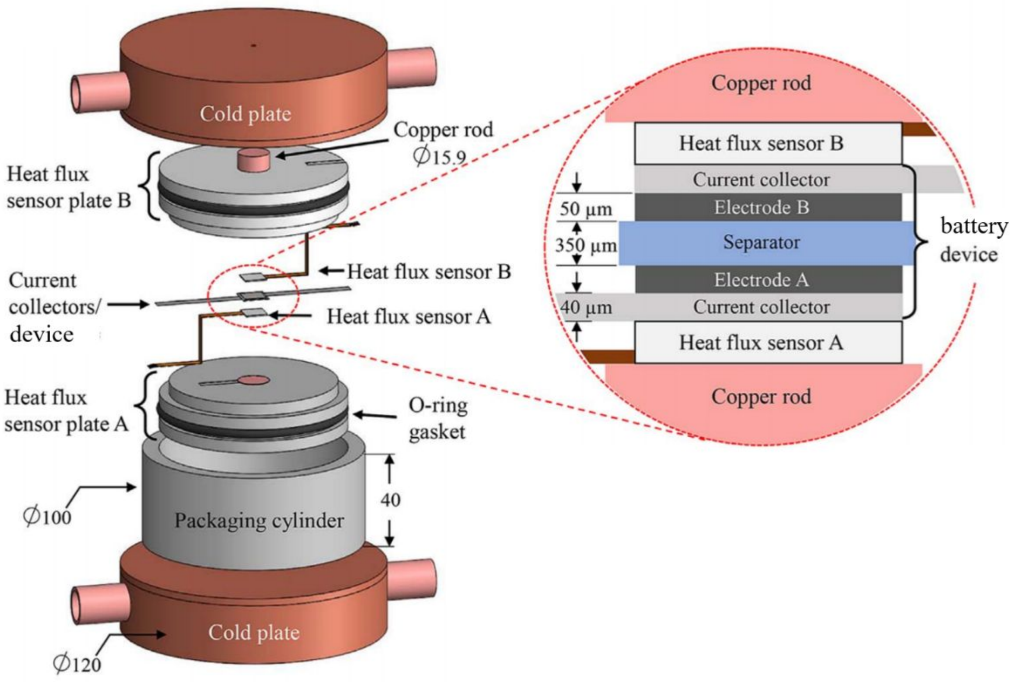

Figure S4. Computer aided design (CAD) model and schematic of the major components of the isothermal calorimeter. Reproduced or adapted with permission from Ref.[27]. Copyright 2018 Elsevier.

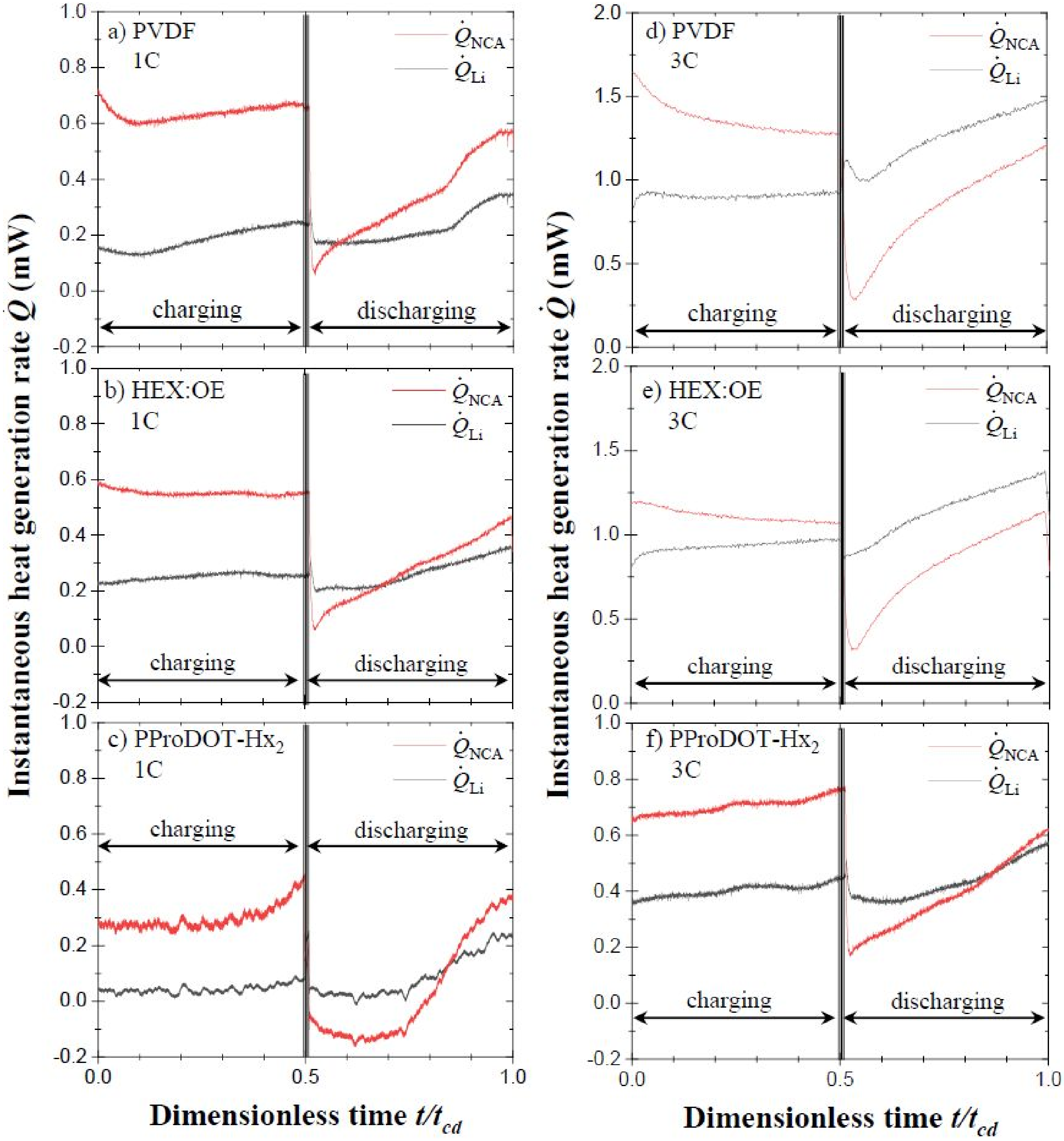

Figure S5. Measured instantaneous heat generation rates $\dot{Q}_{NCA}(t)$ at the NCA electrodes and $\dot{Q}_{Li}(t)$ at the lithium metal electrode averaged over five consecutive cycles as a function of dimensionless time t_{cd} with the potential window ranging from 2.7 V to 4.2 V vs. Li/Li^+ (a, b, c) at C-rates of 1C and (d, e, f) at 3C. Data are shown for NCA electrodes made with polymer binders (a,d) PVDF, (b,e) Hex:OE (75:25), and (c,f) PProDOT-Hx_2.
